# Female fecundity traits in wild populations of African annual fish: the role of the aridity gradient

**DOI:** 10.1002/ece3.2337

**Published:** 2016-07-25

**Authors:** Milan Vrtílek, Martin Reichard

**Affiliations:** ^1^ Institute of Vertebrate BiologyAcademy of Sciences of the Czech RepublicBrnoCzech Republic

**Keywords:** Annual killifish, egg size, interpopulation variation, intrapopulation variability, life expectancy, reproductive allocation

## Abstract

The evolution of life history is shaped by life expectancy. Life‐history traits coevolve, and optimal states for particular traits are constrained by trade‐offs with other life‐history traits. Life histories contrast among species, but may also diverge intraspecifically, at the level of populations. We studied the evolution of female reproductive allocation strategy, using natural populations of two sympatric species of African annual fishes, *Nothobranchius furzeri* and *Nothobranchius orthonotus*. These species inhabit pools in the Mozambican savanna that are formed in the rainy season and persist for only 2–10 months. Using 207 female *N. furzeri* from 11 populations and 243 female *N. orthonotus* from 14 populations, we tested the effects of genetic background (intraspecific lineage) and life expectancy (position on the aridity gradient determining maximum duration of their temporary habitat) on female fecundity traits. First, we found that variation in female body mass was small within populations, but varied considerably among populations. Second, we found that fecundity was largely defined by female body mass and that females spawned most of their eggs in the morning. Third, we found that the trade‐off between egg size and egg number varied among lineages of *N. furzeri* and this outcome has been confirmed by data from two separate years. Overall, we demonstrate that local conditions were important determinants for *Nothobranchius* growth and fecundity and that eggs size in arid region was less limited by female fecundity than in humid region.

## Introduction

Life‐history traits coevolve due to positive feedbacks and specific constraints (trade‐offs), forming complex life‐history strategies (Stearns 1992; Roff and Fairbairn 2007). The evolution of life history under divergent life expectancy (extrinsic mortality) involves trade‐offs between current and future reproduction (Williams 1966; Michod 1979; Kirkwood and Rose 1991). Here, we empirically test how different life expectancy shapes the evolution of female reproductive effort in wild populations.

Short life expectancy is predicted to select for rapid maturation (Kirkwood and Rose 1991; Kozłowski 1992). Females from populations with high predation pressure mature earlier and at smaller size (Reznick et al. 2004), and this negatively affects asymptotic body size even in animals with indeterminate growth as acquired energy is partly reallocated from growth to reproduction (Kozłowski 1992; Heino and Kaitala 1999; Quince et al. 2008). Short life expectancy should also favor maximization of reproductive allocation soon after attaining maturity (Michod 1979; Kindsvater et al. 2011), because the costs of large early‐life reproductive effort (e.g., in terms of survival; Hutchings 1993; Descamps et al. 2006; Boonekamp et al. 2014; Sletvold and Ågren 2015) are unlikely to be encountered. Therefore, populations with a short life expectancy are predicted to have small body size and high relative allocation to reproduction early after maturity (Stearns 1992).

Reproductive investment has to be balanced between offspring quality and quantity (Smith and Fretwell 1974). Increased investment in individual offspring increases its fitness (Bernardo 1996a; Rollinson and Hutchings 2013), but decreases female fecundity (Lim et al. 2014; Stahlschmidt and Adamo 2015). Females have control over the outcome of this parent–offspring conflict, and they tend to maximize their own fitness over the fitness of each of their offspring (Einum and Fleming 2000; Janzen and Warner 2009). This is especially pronounced in unpredictable environments where increased offspring quantity increases the probability that some offspring survives until reproduction (Winemiller and Rose 1993; Morrongiello et al. 2012).

**Figure 1:**
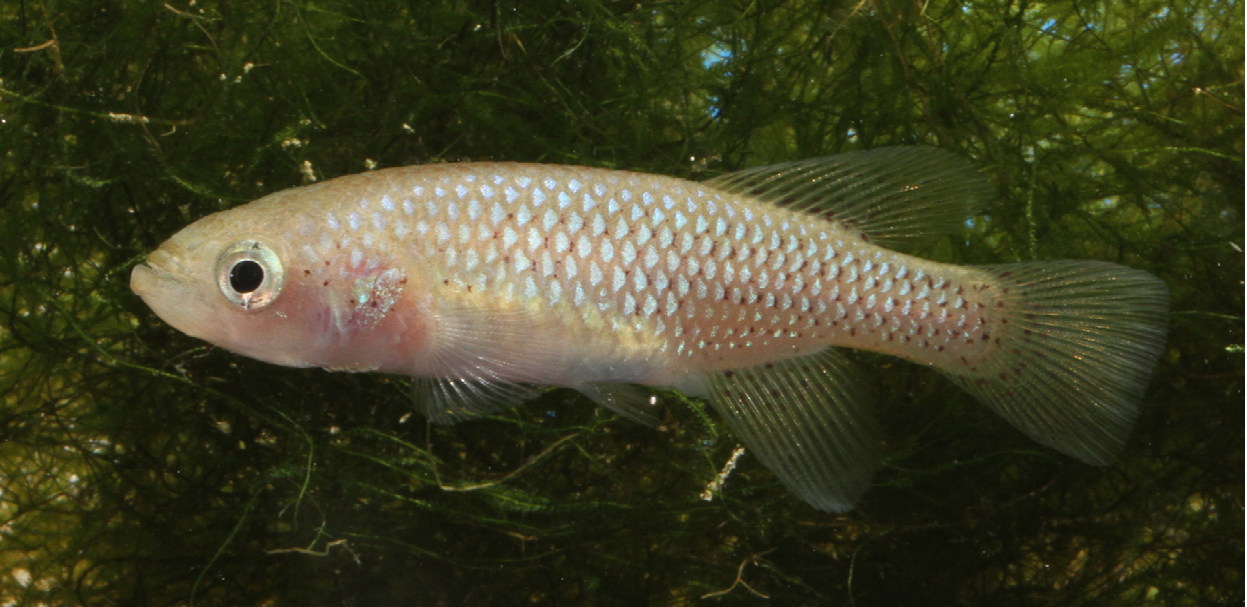
Female *Nothobranchius orthonotus*.

Ephemeral habitats represent a uniquely constrained environment, with a clear upper limit for survival of many resident species when the habitat disappears (Williams 2006). This generates particularly strong selective pressure on reproductive life‐history traits due to the relatively high risk of reproductive failure. Annual fish of the genus *Nothobranchius* (Fig. 1) are adapted to temporary savannah pools of East Africa (Cellerino et al. 2016). The pool desiccation eliminates all adult fish; embryos survive in desiccation‐resistant eggs in the dry pool sediment until the next wet period. Embryo survival over several months of the dry period is enabled by metabolic arrest during diapause (Wourms 1972). *Nothobranchius* fish hatch synchronously when the pool is inundated, producing a single age cohort (Polačik et al. 2011). The young fish grow rapidly and reach sexual maturity 3–5 weeks after hatching (Blažek et al. 2013). After maturity, females lay their eggs daily, distributing them among numerous (20–50), single‐egg clutches spawned several times each day, with multiple males. *Nothobranchius* courtship is simple and spawning is rapid (Haas 1976a; Passos et al. 2015), each spawning act lasting few seconds.

**Figure 2:**
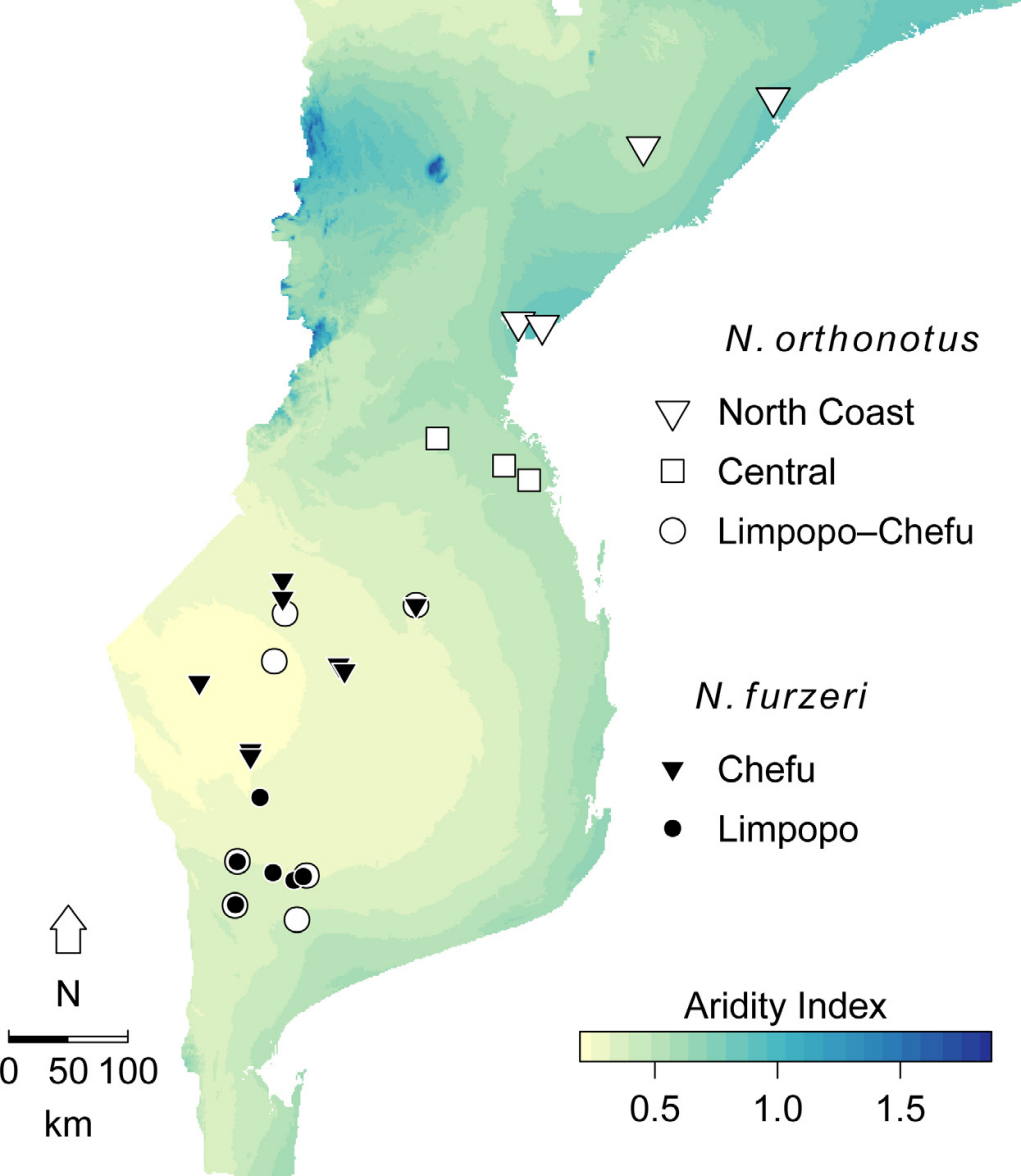
Map of aridity gradient in southern and Central Mozambique. Sampled populations are indicated by filled (*N. furzeri*) or empty (*N. orthonotus*) symbols. Different shape of symbols specifies different mitochondrial lineage in each species.

The climate of Southern and Central Mozambique is characterized by a pronounced gradient of aridity ranging from the relatively humid coastal region to more arid areas inland (Terzibasi Tozzini et al. 2013; Fig. 2). The onset of seasonal rains and time of pool flooding is concordant across the region (Terzibasi Tozzini et al. 2013), but differences in precipitation and evapotranspiration lead to variation in pool duration (desiccation) along the gradient (Terzibasi Tozzini et al. 2013). *Nothobranchius* populations are highly genetically structured (Bartáková et al. 2013, 2015), allowing potential evolution of different life‐history optima. Hence, populations across the aridity gradient differ in their life expectancy and interpopulation lifespan differences were recorded in captivity (Terzibasi et al. 2008).

We test the effect of different life expectancy on interpopulation variation in fecundity‐related life‐history traits using *Nothobranchius furzeri* and *Nothobranchius orthonotus*, two species sympatric across a large part of Southern and Central Mozambique. The species co‐occur syntopically throughout *N. furzeri*'s range, with *N. orthonotus* range extending further northward (Fig. 2; Bartáková et al. 2015; Vrtílek and Reichard 2016). *Nothobranchius orthonotus* occurs at lower population densities and attains larger asymptotic size than *N. furzeri* (Reichard et al. 2009; as *N. kuhntae* in Genade et al. 2005), although their ecological niche (habitat use and diet) largely overlaps (Polačik and Reichard 2010; Polačik et al. 2014a).

We hypothesized that natural selection would maximize reproductive success under the variable limits of habitat existence. Specifically, we tested two main predictions. First, females from populations with short life expectancy would invest relatively more energy into current reproduction (ovary mass and number of eggs). Second, females from populations with short life expectancy would invest in offspring quantity rather than quality, producing more eggs of a smaller size compared to populations with longer life expectancy. In addition, we predicted that the populations of *N. orthonotus* would have more pronounced differences in fecundity traits between the extremes of the life expectancy gradient as its range extends over a larger part of the gradient.

## Methods

### Sample and data collection

We have sampled populations of two *Nothobranchius* species along gradient of life expectancy in Central and Southern Mozambique (Terzibasi Tozzini et al. 2013). The life expectancy gradient was defined using aridity index, a ratio between the mean annual precipitation and mean annual potential evapotranspiration based on 1950–2000 averaged data (Trabucco and Zomer 2009; Fig. 2). The aridity index corresponded well to our records on actual desiccation of individual *Nothobranchius* pools (inundated vs. desiccated) across the study area based on five visits at the end of rain season (Figure S1).

We collected females from 11 populations of *N. furzeri* in 2011 (25 February–13 March) and 14 populations of *N. orthonotus* in 2012 (24 February–13 March; Fig. 2, Table S1). We targeted a sample of 20 females per population; a haphazardly chosen subsample was taken when more female were collected. Fish were sacrificed with an overdose of clove oil, stored in 10% formaldehyde and transported to the Institute of Vertebrate Biology of ASCR in Brno, Czech Republic, for further analyses (for collection and export permits see Acknowledgments).

Females were dissected and weighed to record eviscerated body and ovary mass to the nearest 0.001 g (body mass: Wd, ovary mass: Wg). After ovary dissection, the number of mature oocytes was counted (number of eggs: No). In *Nothobranchius*, mature oocytes (eggs) are large and translucent and can be readily distinguished from opaque immature oocytes (Haas 1976a; Vrtílek and Reichard 2015). The eggs were photographed under a dissecting microscope at 4× magnification (Olympus SZX10, Olympus Corporation, Shinjuku, Tokyo, Japan) with digital camera (μEye‐1540C). Egg diameter was measured in pixels along its longest axis using ImageJ software ver. 1.46j (Rasband 2012) and converted to the nearest 0.001 mm using a reference microglass (egg size: Dia). Summary data for individual populations are given in Table S1.

The sampled populations clustered into mitochondrial lineages (2 in *N. furzeri* and 3 in *N. orthonotus*, Fig. 2; Bartáková et al. 2013, 2015). In total, we analyzed 141 *N. furzeri* females belonging to Chefu and 66 to Limpopo mitochondrial lineage. In *N. orthonotus*, 113 females were from Limpopo–Chefu, 49 from Central, and 81 from North Coast mitochondrial lineage. To test the reliability of the final model for *N. furzeri*, we also analyzed data on six populations (117 females) of *N. furzeri* collected in 2012 (24 February–1 March). Only populations from Limpopo mitochondrial lineage were available, because pools in the Chefu were mostly dry during our visit in 2012. In total, we measured 3871 (year 2011) and 1625 eggs (year 2012) of *N. furzeri* females, and 1212 eggs from *N. orthonotus* females.

### Sampling time

Data from captive fish indicated that *Nothobranchius* females ovulate eggs overnight, commence spawning 3 h after sunrise and then spawn throughout the day, with a peak at noon (Haas 1976b). Analysis of number of eggs in this study revealed that there was a strong negative correlation between time when sampling was conducted (Sampling Time) and proportion of females with mature oocytes in their ovaries (Figure S2). Later sampling time also increased the intrapopulation variation of number of eggs (data not shown). This indicated that a considerable number of spawning events occurred in the morning. Sampling of all the populations in early morning was not logistically feasible; therefore, we did not analyze number of eggs as a parameter of female reproductive allocation. Note, however, that number of eggs was used as a covariate in the analysis of egg size. For ovary mass, we assumed a continuous production of immature oocytes due to intense and continuous reproduction by *Nothobranchius* females (Vrtílek and Reichard 2015). Consequently, we analyzed ovary mass also with respect to the potential confounding effect of sampling time.

### Data analysis

In the statistical analysis, we followed an information‐theoretic (I‐T) approach for selecting the best approximating model for our data (Burnham and Anderson 2002). We built a set of candidate models that represented hypotheses for each response variable (body mass, ovary mass and egg size) including the null models (intercept only; Tables 1 and 2). Body mass was used as a covariate in the analysis of ovary mass because fecundity in fish is largely dependent on female body size (Wootton and Smith 2015; Table 1A). In the egg size analysis, the covariates ovary mass and number of eggs accounted for energy allocated into reproduction (Wg) and its partitioning among specific number of eggs (No) (i.e., controlling for offspring number/size trade‐off, Smith and Fretwell (1974); Table 2).

**Table 1:** Outcome of model selection in analysis of ovary mass for *N. furzeri* (A) and *N. orthonotus* (B). Models with considerable support (∆AIC_c_ <2 from the model with the lowest AIC_c_ value) are emphasized in bold. Fixed terms included body mass (Wd) (log‐transformed), aridity index (AI), genetic structure (affiliation to mitochondrial lineage) (GEN), and Sampling time (TIME)

Model NF	df	logLik	∆AIC_c_	Akaike weight	Fixed terms
(A)					
**m6a**	**7**	−**24.30**	**0.00**	**0.361**	**Wd** + **AI** + **TIME** + **TIME** × **Wd**
**m6b**	**7**	−**24.70**	**0.81**	**0.241**	**Wd** + **GEN** + **TIME** + **TIME** × **Wd**
**m7a**	**8**	−**23.93**	**1.43**	**0.176**	**Wd** + **AI** + **TIME** + **AI** × **Wd** + **TIME** × **Wd**
m7b	8	−24.67	2.92	0.084	Wd + GEN + TIME + GEN × Wd + TIME × Wd
m5a	7	−26.41	4.22	0.044	Wd + AI + TIME + AI × Wd
m3c	5	−28.97	5.07	0.029	Wd + TIME
m4a	6	−28.10	5.47	0.023	Wd + AI + TIME
m2	4	−30.94	6.93	0.011	Wd
m4b	6	−28.90	7.05	0.011	Wd + GEN + TIME
m5b	7	−27.83	7.07	0.011	Wd + GEN + TIME + GEN × Wd
m3a	5	−30.63	8.40	0.005	Wd + AI
m3b	5	−30.94	9.03	0.004	Wd + GEN
m1	3	−82.00	106.96	0.000	~

Model NO	df	logLik	∆AIC_c_	Akaike weight	Fixed terms
(B)					
**m2**	**4**	−**175.27**	**0.00**	**0.223**	**Wd**
**m3a**	**5**	−**174.45**	**0.46**	**0.178**	**Wd** + **AI**
**m3b**	**6**	−**173.78**	**1.21**	**0.122**	**Wd** + **GEN**
**m5b**	**9**	−**170.57**	**1.21**	**0.122**	**Wd** + **GEN** + **TIME** + **GEN** × **Wd**
m3c	5	−175.26	2.07	0.079	Wd + TIME
m4a	6	−174.45	2.55	0.062	Wd + AI + TIME
m5a	7	−173.57	2.91	0.052	Wd + AI + TIME + AI × Wd
m4b	7	−173.61	3.00	0.050	Wd + GEN + TIME
m7b	10	−170.52	3.28	0.043	Wd + GEN + TIME + GEN × Wd + TIME × Wd
m6a	7	−174.26	4.28	0.026	Wd + AI + TIME + TIME × Wd
m7a	8	−173.32	4.54	0.023	Wd + AI + TIME + AI × Wd + TIME × Wd
m6b	8	−173.57	5.04	0.018	Wd + GEN + TIME + TIME × Wd
m1	3	−232.07	111.54	0.000	~

All models included random intercept for a population.

**Table 2:** Outcome of model selection in egg size analysis for *N. furzeri* (A) and *N. orthonotus* (B). Models with considerable support (∆AIC_c_ <2 from the model with the lowest AIC_c_ value) are emphasized in bold. Fixed terms included ovary mass (Wg) (log‐transformed), number of eggs (No), aridity index (AI), and genetic structure (affiliation to mitochondrial lineage) (GEN)

Model NF	df	logLik	∆AIC_c_	Akaike weight	Fixed terms
(A)					
**m5b**	**11**	**5688.46**	**0.00**	**0.99**	**Wg** + **No** + **Wg** × **No** + **GEN** + **Wg** × **GEN** + **No** × **GEN** + **Wg** × **No** × **GEN**
m3	7	5678.53	11.82	0.00	Wg + No + Wg × No
m4b	8	5679.50	11.88	0.00	Wg + No + Wg × No + GEN
m4a	8	5678.57	13.75	0.00	Wg + No + Wg × No + AI
m5a	11	5680.39	16.14	0.00	Wg + No + Wg × No + AI + Wg × AI + No × AI + Wg × No × AI
m1	4	5662.63	37.59	0.00	~
m2b	5	5663.35	38.16	0.00	GEN
m2a	5	5662.89	39.07	0.00	AI

Model NO	df	logLik	∆AIC_c_	Akaike weight	Fixed terms
(B)					
**m4b**	**9**	**1544.09**	**0.00**	**0.35**	**Wg** + **No** + **Wg** × **No** + **GEN**
**m4a**	**8**	**1543.07**	**0.01**	**0.35**	**Wg** + **No** + **Wg** × **No** + **AI**
m3	7	1540.90	2.31	0.11	Wg + No + Wg × No
m5a	11	1544.90	2.45	0.10	Wg + No + Wg × No + AI + Wg × AI + No × AI + Wg × No × AI
m2a	5	1538.20	3.68	0.06	AI
m2b	6	1538.06	5.98	0.02	GEN
m1	4	1535.87	6.32	0.01	~
m5b	15	1546.11	8.21	0.01	Wg + No + Wg × No + GEN + Wg × GEN + No × GEN + Wg × No × GEN

All models included nested random intercept for female within population.

The main explanatory factors tested were aridity index and genetic structure. Aridity index approximated the expected lifespan along the climatic gradient, and genetic structure controlled for independent evolution of traits and their trade‐offs within mitochondrial lineages (Bartáková et al. 2013, 2015; Valenzano et al. 2015). These two factors were neither exclusive nor fully crossed and represented different partitioning of the life expectancy gradient. Aridity index was a continuous variable, while individual mitochondrial lineages inhabit regions with specific aridity, thereby genetic structure divided the gradient into 2 (in *N. furzeri*) or 3 (in *N. orthonotus*) categories. Hence, we tested the effect of these two explanatory factors as alternative competing hypotheses.

Potential confounding effects were accounted for by including sampling date (number of days after sampling of the first population) in body mass analysis and sampling time (time of the day when population was sampled, rounded to a quarter of an hour) in ovary mass analysis (Table 1). Intrapopulation variation was controlled by the random effect of population. Similarly, the random effect of female ID nested in population (Population/Female ID) was included in the analysis of egg size (Table 2). Body mass and ovary mass were naturally log‐transformed to linearize their relationship (Bolker et al. 2009) and to remove heteroscedasticity. All the continuous explanatory variables were centered to facilitate interpretation of model coefficient estimates (Schielzeth 2010; Grueber et al. 2011).

To fit the candidate models, we used linear mixed‐effects modeling from the “nlme” package (Pinheiro et al. 2015) in R software ver. 3.1.3 (R Core Development Team 2015). The models were compared using a second‐order version of the akaike information criterion suited to small sample size – AIC_c_. Models within AIC_c_ difference (∆AIC_c_) of 9–11 were kept in the set of plausible models (Burnham et al. 2011). We used akaike weights to compare models in the candidate model set with values exceeding 0.9 indicating that reliable inference can be made based on that single best model (Burnham and Anderson 2002). When the akaike weight was lower, model averaging was used (Burnham and Anderson 2002; Burnham et al. 2011; Grueber et al. 2011). Averaging was applied only over models containing the focal term to obtain the coefficient and associated error estimates (i.e., conditional model averaging, Burnham and Anderson 2002). The sum of akaike weights of models containing particular variables gave the relative variable importance (RVI), a measure of how important a particular variable is in the given set of models. We then quantified model goodness‐of‐fit ($R_{\mathrm{GLMM}}^{2}$) for the fixed part of a model ($R_{\mathrm{GLMM}}^{2}$ marginal) and for the complete model including its random effects ($R_{\mathrm{GLMM}}^{2}$ conditional; Nakagawa and Schielzeth 2013). This enabled us to compare the amount of explained variability among models and between the fixed and random parts of a specific model. All these parameters were extracted using the “MuMIn” package (Bartoń 2015). Finally, random‐effect intraclass correlation coefficient specified population or female consistency in life‐history traits (Nakagawa and Schielzeth 2010).

## Results

### Body mass: strong interpopulation variation

**Figure 3:**
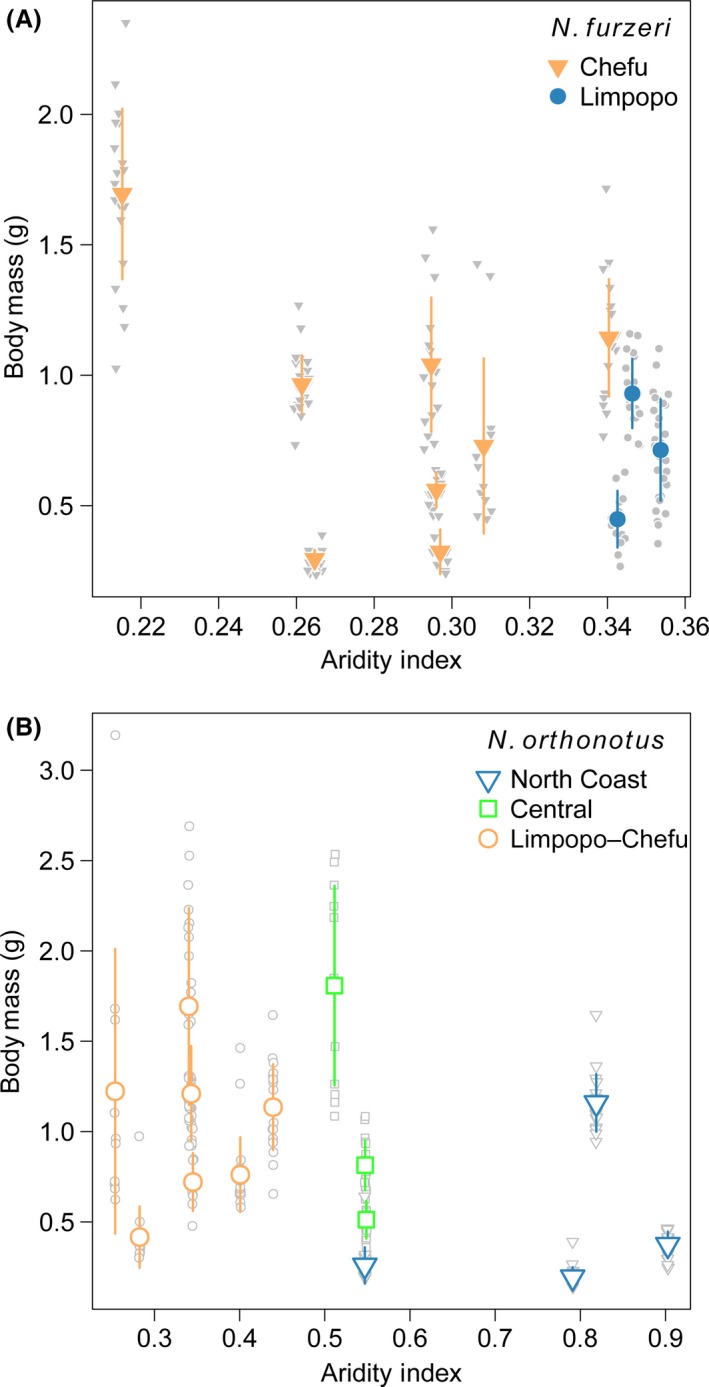
Body mass variability among sampled wild populations of *N. furzeri* (A) and *N. orthonotus* (B). Different shape of symbols specifies different mitochondrial lineage in each species. Smaller gray points show values for individual females, and large point (error line) indicates population mean (SD).

In both species, female body mass variation was small within populations but large among populations (Fig. 3). Interpopulation variation in body mass was not explained by any of the measured explanatory variables. The models with aridity index, genetic structure, or sampling date received support similar to the null model (maximum ∆AIC_c_ = 2.02 and 3.92, in *N. furzeri* and *N. orthonotus*, respectively). The amount of variation explained by the individual fixed factors was small ($R_{\mathrm{GLMM}}^{2}$ marginal = 0.4–2.8% in *N. furzeri* and 0.6–28.6% in *N. orthonotus*), but the whole model (including population as a random effect) explained a large proportion of the variation in body mass ($R_{\mathrm{GLMM}}^{2}$ conditional = 86.4–87.6%).

### Residual variation in ovary mass was not affected by the aridity index or genetic structure

Ovary mass was primarily dependent on female body mass; body mass substantially improved the model fit compared to the null model (∆AIC_c_ for models with and without body mass >95 in both species). No additional explanatory factors improved the model fit substantially (Table 1).

In *N. furzeri*, models that included the effect of sampling time on ovary mass through body mass (m6a, m6b, m7a; Table 1A) were better supported than other candidate models. The best models contained sampling time (RVI = 0.98) and its interaction with body mass (RVI = 0.86). The effect of late sampling time on ovary mass was stronger in larger *N. furzeri* females (negative coefficient of the sampling time × body mass; Table S2A). In *N. orthonotus*, high model selection uncertainty prevented reduction of the number of plausible models (∆AIC_c_ among non‐null models = 5.04; Table 1B) and model averaging generated high error estimates suggesting weak approximating power in the explanatory factors (Table S2B). This indicated that the data were not adequate for reaching strong inference.

### Trade‐off between egg size and their number differed among intraspecific lineages

Egg size covaried negatively with number of eggs in both species. Inclusion of genetic structure affected the strength of this relationship in *N. furzeri*. In *N. orthonotus,* there was an additive tapering effect of the aridity index and genetic structure on egg size.

**Figure 4:**
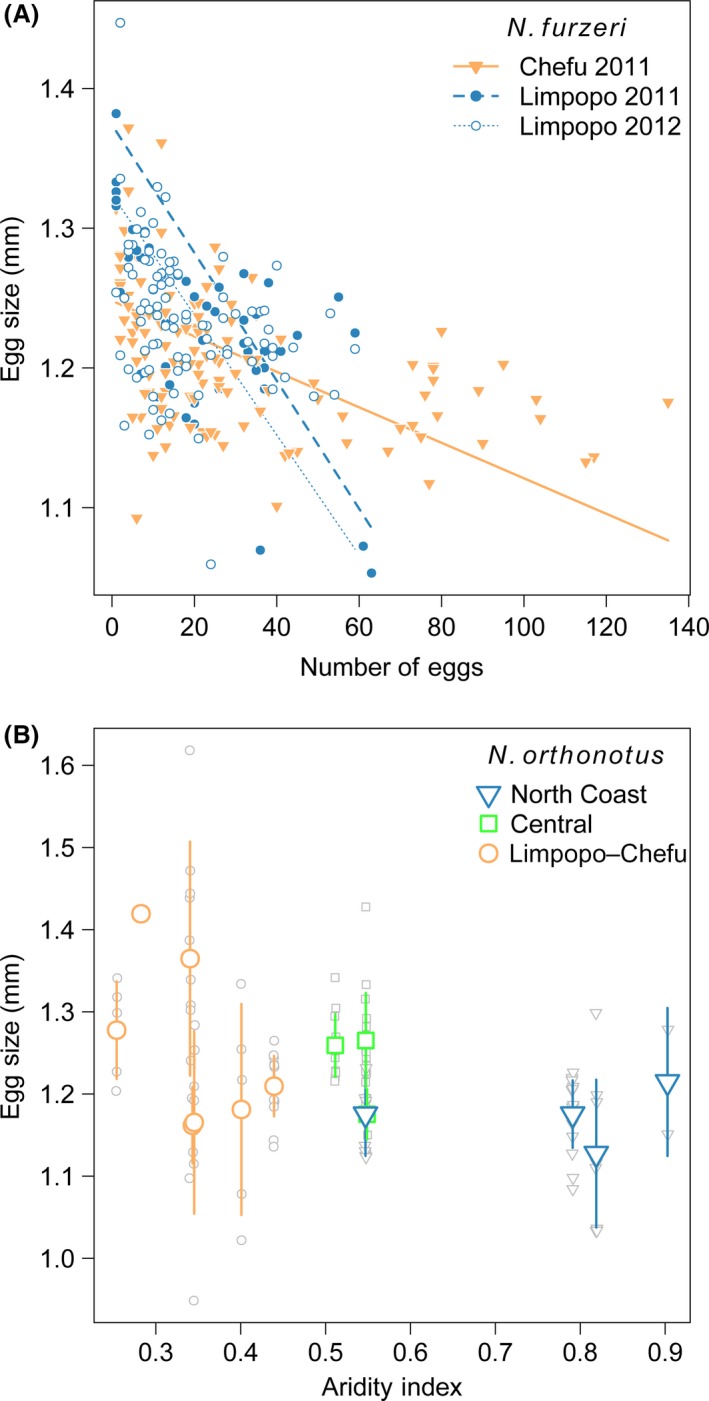
Relationship between egg size and number of eggs in *N. furzeri* females (A) and with respect to aridity index in *N. orthonotus* populations (B). Smaller gray points show values for individual females, and large point (error line) indicates population mean (SD).

The analysis of egg size in *N. furzeri* revealed a single superior model including interaction between the trade‐off measure (number of eggs × ovary mass) and genetic structure (m5b, ∆AIC_c_ = 11.82, Akaike weight = 0.99; Table 2A). In addition to differences in egg size among mitochondrial lineages of *N. furzeri* per se (Table S3A), the lineages also differed in the strength of the trade‐off between egg size and the number of eggs. In the Limpopo lineage, a higher number of eggs was associated with a greater decrease in egg size than in the Chefu lineage (Table S3A, Fig. 4A). Model validation with the 2012 dataset yielded very similar estimates of model coefficients (Table S3B), confirming that this relationship was not a sampling or analytical artifact.

In contrast to *N. furzeri*, there was profound model selection uncertainty in egg ize analysis in *N. orthonotus* (maximum ∆AIC_c_ = 8.21, Table 3B). Averaged coefficients suggested a tendency toward smaller egg size in the humid part of the aridity gradient (estimate of effect of aridity index [±SE]: −0.186 ± 0.129) and in the North Coast mitochondrial lineage (1.188 ± 0.044) compared to the other two lineages (egg size estimate for Central and Chefu–Limpopo lineage [±SE]: 1.286 ± 0.040 and 1.275 ± 0.033, respectively) (Fig. 4B, Table S3C).

## Discussion

Females of both *Nothobranchius* species displayed profound interpopulation variation in their life‐history traits. Their body mass was population specific, with a high similarity among females from the same population although the variation was not related to life expectancy. The number of eggs in ovaries and ovary mass among populations decreased with sampling time over the course of the day and did not correspond to life expectancy. Egg size depended on the number of eggs a female possessed in her ovaries, but differed between mitochondrial lineages in both *Nothobranchius* species. Mitochondrial lineages of *N. furzeri* differed in the strength of the trade‐off between egg size and number; the lineage from the region with longer expected lifespan (the humid region) showed a steeper decrease in egg size as a function of egg number compared to females from the arid region. In *N. orthonotus*, relatively smaller eggs tended to be produced in the mitochondrial lineage inhabiting the humid region compared to the two lineages from more arid areas.

Smaller female body mass can be predicted in arid regions (with short life expectancy) as a result of selection for early maturity. However, we found no relationship between life expectancy and female body mass in the wild populations of either annual fish species. Early maturity may be achieved through faster growth when resources are abundant (Stearns and Koella 1986; Blanckenhorn 2000). Given that realized growth consists of additive genetic and environmental effects (Arendt 1997), any genetic variation in growth rates among populations was likely overridden by resource availability in particular populations. Resource availability may be dynamic in the temporary savannah pools (Meintjes 1996) and vary among pools (Polačik and Reichard 2010). Moreover, the growth of *Nothobranchius* fishes is very plastic (Blažek et al. 2013; Vrtílek and Reichard 2015). Therefore, density‐dependent effect likely overridden any genetic effects related to the position on life expectancy gradient and our data corroborate that adaptive divergence in growth rates can only be detected in common garden environments (Bronikowski 2000; Ab Ghani et al. 2012; Tibblin et al. 2015).

We predicted higher fecundity in populations from the arid region due to their higher allocation to reproduction (Kirkwood and Rose 1991), but this was not supported by our data. A strong confounding effect of sampling time was detected in both study species and affected both the number of eggs and ovary mass. In captivity, *Nothobranchius* females release a small batch of eggs per spawning multiple times per day (Haas 1976b; Polačik and Reichard 2011). Haas (1976b) reported peak of spawning activity in the closely related, captive *N. guentheri* at noon, and our observational data suggested the same for captive *N. furzeri* and *N. orthonotus*. In the wild, spawning likely occurred much earlier. While more than 90% of *N. furzeri* females still possessed some eggs in their ovaries at 10:00, over 50% of *N. orthonotus* females had spent all their eggs by that time (Figure S2). This indicates much earlier spawning under natural conditions than in captivity and faster egg depletion in *N. orthonotus* than in *N. furzeri*. Further, ovary mass declined more steeply in larger *N. furzeri*, indirectly suggesting that, over the course of the day, larger females may have spawned earlier or more rapidly than smaller females.

Our data demonstrate that *Nothobranchius* females are an extreme case of batch spawners (Wootton and Smith 2015); females spawn continuously during the day (Figure S2), with a peak in the morning, and ovulate eggs overnight. Continuous daily reproduction seems important for annual fish that face high mortality risk due to the erratic character of their habitat. Neotropical annual killifish *Cynopoecilus melanotaenia* show a similar pattern of mature oocytes recruitment under natural conditions (Arenzon et al. 1999). This reproductive strategy, however, is not restricted to annual fish. In Japanese medaka (*Oryzias latipes*), females are reported to spawn daily in captivity when fed appropriately (Bryant and Grant 1995). On the other hand, lower reproductive effort was reported in female Trinidadian stream killifish *Rivulus hartii* that produced on average 28 eggs over a 2‐week period (Walsh and Reznick 2008). The same number of eggs is regularly produced during a 2‐h period in captive *Nothobranchius furzeri* and *N. orthonotus* after 48‐h separation from males (Blažek et al. 2013; Polačik et al. 2016). When egg turnover is so high, the data on energy allocation from the wild have to be interpreted carefully. Several factors may affect the interpretation, such as resource acquisition (van Noordwijk and de Jong 1986; Messina and Fry 2003; Uller and Olsson 2005) or male density (Reichard et al. 2014). These potentially confounding factors cannot be controlled during field sampling. In conclusion, reliable inferences on the evolution of reproductive allocation strategy among *Nothobranchius* populations across a gradient of life expectancy cannot be made from wild fish and common garden experiments conducted under controlled environmental conditions are needed.

We predicted smaller eggs in females from short life expectancy populations as an adaptation to maximize fecundity under the restricted time available for reproduction. As expected, egg size was negatively associated with egg number due to an inherent trade‐off between size and number of eggs. In *N. furzeri*, this trade‐off varied among mitochondrial lineages. There was a steeper decrease in egg size with increasing egg number in the longer life expectancy populations (Limpopo lineage). In populations with shorter life expectancy (Chefu lineage), the trade‐off was not evident in highly fecund females (40–140 eggs). In *N. orthonotus*, the strength of the egg size/number relationship was similar among the mitochondrial lineages, but we recorded lineage‐specific egg size per standardized number of eggs. Contrary to our prediction, the eggs of females from the long life expectancy mitochondrial lineage (North Coast lineage, the most humid area) were relatively smaller than those of females from the more arid part of the species distribution (Central and Limpopo–Chefu lineages).

The fact that females from the environment with shorter expected lifespan tended to produce relatively larger eggs (*N. orthonotus*) at least at higher fecundities (*N. furzeri*) suggests a potential constraint on egg size in *Nothobranchius* fish. Reduction of per offspring expenditure and consequent fecundity increase is only possible when conditions are favorable for offspring survival (Bernardo 1996b; Fox and Czesak 2000). Selection favors few large offspring in harsh environments (Smith and Fretwell 1974) because large eggs produce larger offspring that cope better with challenging environmental conditions (Einum and Fleming 1999). There are at least two possible reasons why larger eggs may be beneficial in *Nothobranchius*; both are related to the availability of energetic resources. First, in an environment with short life expectancy, fast initial growth and early maturation is critical for successful reproduction. This is comparable to situations where even perennial animals face seasonal stress. At higher latitudes and altitudes, where the breeding season is short, amphibians lay larger eggs that give rise to larger offspring who achieve metamorphosis faster than conspecifics in areas with longer breeding seasons (Morrison and Hero 2003). Thus, producing offspring with additional energetic resources can be adaptive despite higher parental cost per individual offspring (Smith and Fretwell 1974). In a species of bdelloid rotifer, larger energetic reserves of bigger eggs fuel rapid initial growth and help offspring to mature quickly (Santo et al. 2001). *Nothobranchius* life is exceptionally fast, and they are able to mature and become sexually active 3 weeks after hatching in favorable conditions (Blažek et al. 2013). In *N. furzeri*, high energetic reserves and small hatchling length are traits characteristic for a composite “fast” phenotype (Polačik et al. 2014b). Under standardized conditions, these “fast” fish matured 2 days earlier than the “slow” fish that hatched with smaller reserves but at a larger size (Polačik et al. 2014b).

Larger eggs may be favored in *Nothobranchius* populations due to the temporary character of the *Nothobranchius* habitat. Long period of habitat desiccation (several months) represents a survival challenge for embryos during the dry season when persist in a diapausing stage in the sediment. Even though energy expenditure is severely limited during diapause (Podrabsky and Hand 1999), any additional energetic resources may increase their survival in transient periods when embryos are outside diapause and awaiting favorable conditions for hatching. Larger eggs may facilitate longer survival in diapause; metabolic processes are lowered during diapause, but there is still measurable metabolic activity (Podrabsky and Hand 1999), and *Nothobranchius* embryos can persist in diapause for several years (Polačik et al. 2016). The potential role of these two aspects (rapid initial growth and prolonged survival through the embryonic stage related to egg resources) of *Nothobranchius* life history in the evolution of its egg size remains to be experimentally examined.

## Conclusions

We tested the effect of life expectancy on female life‐history traits in natural populations of two sympatric species of annual fish in the genus *Nothobranchius*.

1. We found that female body mass was similar within populations, but varied strongly among populations. This implies that local conditions and growth plasticity are important determinants for *Nothobranchius* growth, overriding any potential genetic effects.
2. The fecundity of *Nothobranchius* females was largely defined by their body mass, but populations sampled later in a day had already spawned most of their eggs. This indicates that the intensive reproduction of *Nothobranchius* fish observed in captivity is also common in wild populations. Ultimately, this prevented inference of any general conclusions on the evolution of reproductive allocation under divergent life expectancy.
3. We identified a potential constraint on *Nothobranchius* egg size. We propose two explanations of its source related to the amount of energetic reserves of the egg – speed of early posthatching growth and ability of prolonged survival of diapausing embryos during the dry period. These hypotheses remain to be tested.

## Conflict of Interest

None declared.

## Supporting information


**Figure S1.** Logistic regression of *Nothobranchius* pool desiccation and position of the pool on the aridity gradient expressed by aridity index.
**Figure S2.** Effect of sampling time on female spawning status.
**Table S1.** Population characteristics including summary of female life‐history traits (mean ± SD) for the two studied *Nothobranchius* species.
**Table S2.** Model‐averaged coefficient estimates for predictors of ovary mass.
**Table S3.** Coefficient estimates for predictors of egg size.
