# Supplementary material for: Female fecundity traits in wild populations of African annual fish: the role of the aridity gradient
Source: Ecol Evol. 2016 Jul 25;6(16):5921–31. doi: 10.1002/ece3.2337 (PMC4983602; doi:10.1002/ece3.2337)
Supplement: Supplementary file 1 — Figure S1. Logistic regression of Nothobranchius pool desiccation and position of the pool on the aridity gradient expressed by aridity index. Figure S2. Effect of sampling time on female spawning status. Table S1. Population characteristics including summary of female life‐history traits (mean ± SD) for the two studied Nothobranchius species. Table S2. Model‐averaged coefficient estimates for predictors of ovary mass. Table S3. Coefficient estimates for predictors of egg size. [file ECE3-6-5921-s001.docx]

**Appendix**

**Figure S1.** Logistic regression of *Nothobranchius* pool desiccation and position of the pool on the aridity gradient expressed by Aridity Index.

**Figure S2.** Effect of sampling time on female spawning status.

**Table S1.** Population characteristics including summary of female life-history traits (mean±SD) for the two studied *Nothobranchius* species.

**Table S2.** Model-averaged coefficient estimates for predictors of Ovary Mass.

**Table S3.** Coefficient estimates for predictors of Egg Size.

Figure S1. Logistic regression of *Nothobranchius* pool desiccation and position of the pool on the aridity gradient expressed by Aridity Index. The model fits describes increasing probability that the pool still will be inundated at the end of rainy season with increasing Aridity Index. The estimate of Aridity Index effect is 3.641±0.642 (est.±SE); *F* = 32.22, *z*-value = 5.67, *P* < 0.001). Year ID (5 years) and Pool ID (465 records of 137 different pools) were used as random factors in the generalized mixed-effects model. The line was fitted by logistic regression formula y = 1/(1+exp(-(-0.803+3.641*x)). Inundated pools are indicated by filled points, desiccated pools are marked by empty points.


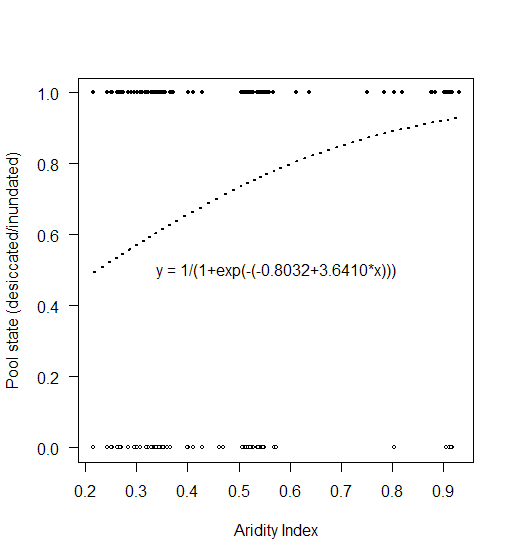


Figure S2. Effect of sampling time on female spawning status. The lines were fitted by 1/(1+exp(-(5.2245-0.4814*x), 1/(1+exp(-(7.86870-0.50545*x) and 1/(1+exp(-(5.4657-0.3705*x) for *N. orthonotus*, *N. furzeri* 2011 and *N. furzeri* 2012, respectively.


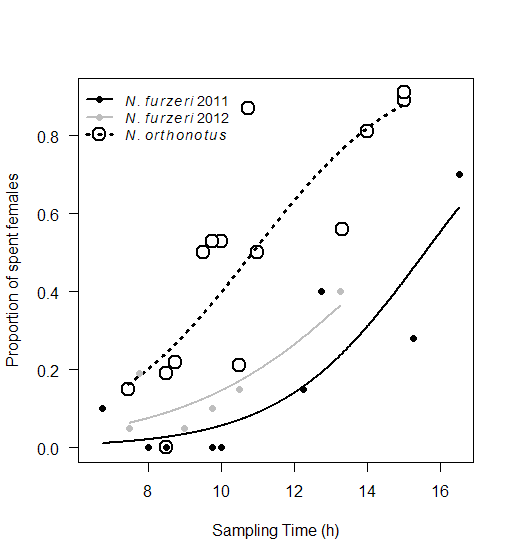


Table S1. Population characteristics including summary of female life-history traits (mean±SD) for the two studied *Nothobranchius* species.

| Species | Population | Longitude | Latitude | Mitochondrial Lineage | Aridity Index | Sampling Date | Sampling Time | Number of females | Proportion of spent females | Body Mass (mg) | Ovary Mass (mg) | Number of Eggs | Egg Size (mm) |
| --- | --- | --- | --- | --- | --- | --- | --- | --- | --- | --- | --- | --- | --- |
| *N. furzeri* 2011 | L043 | 32.53 | -23.3 | Chefu | 0.261 | 28.2.2011 | 15:15 | 25 | 0.28 | 0.965±0.111 | 0.067±0.015 | 8.8±10.0 | 1.256±0.068 |
|  | L053 | 32.95 | -24.37 | Limpopo | 0.354 | 27.2.2011 | 16:30 | 30 | 0.70 | 0.713±0.194 | 0.033±0.008 | 0.6±1.4 | 1.293±0.046 |
|  | L121 | 32.97 | -24.35 | Limpopo | 0.343 | 13.3.2011 | 8:00 | 16 | 0.00 | 0.448±0.109 | 0.048±0.022 | 14.8±8.4 | 1.210±0.073 |
|  | L220 | 32.8 | -22.02 | Chefu | 0.295 | 3.3.2011 | 8:30 | 15 | 0.00 | 1.040±0.257 | 0.092±0.039 | 33.9±19.4 | 1.170±0.058 |
|  | L222 | 32.8 | -21.87 | Chefu | 0.308 | 11.3.2011 | 8:00 | 12 | 0.00 | 0.729±0.336 | 0.065±0.029 | 25.6±14.1 | 1.164±0.055 |
|  | L322 | 32.53 | -23.35 | Chefu | 0.265 | 28.2.2011 | 12:45 | 20 | 0.40 | 0.295±0.035 | 0.015±0.005 | 2.8±2.9 | 1.237±0.073 |
|  | L401 | 32.42 | -24.23 | Limpopo | 0.346 | 25.2.2011 | 10:00 | 20 | 0.00 | 0.930±0.133 | 0.101±0.020 | 38.5±12.3 | 1.198±0.083 |
|  | L413 | 32.1 | -22.73 | Chefu | 0.215 | 1.3.2011 | 10:00 | 20 | 0.00 | 1.695±0.327 | 0.220±0.049 | 83.8±26.4 | 1.170±0.058 |
|  | L418 | 33.32 | -22.63 | Chefu | 0.297 | 2.3.2011 | 10:00 | 10 | 0.00 | 0.323±0.086 | 0.029±0.012 | 10.8±6.4 | 1.226±0.055 |
|  | L419 | 33.27 | -22.58 | Chefu | 0.296 | 2.3.2011 | 12:15 | 20 | 0.15 | 0.560±0.067 | 0.049±0.011 | 17.6±9.3 | 1.193±0.057 |
|  | L423 | 33.92 | -22.08 | Chefu | 0.340 | 4.3.2011 | 6:45 | 20 | 0.05 | 1.143±0.225 | 0.081±0.034 | 20.9±13.1 | 1.213±0.068 |
|  |  |  |  |  |  |  |  |  |  |  |  |  |  |
| *N. furzeri* 2012 | L008 | 32.61 | -23.69 | Limpopo | 0.354 | 1.3.2012 | 7:30 | 21 | 0.05 | 0.549±0.039 | 0.081±0.012 | 36.1±13.6 | 1.224±0.031 |
|  | L053 | 32.95 | -24.37 | Limpopo | 0.288 | 25.2.2012 | 10:30 | 20 | 0.15 | 0.322±0.043 | 0.024±0.007 | 6.8±5.0 | 1.216±0.044 |
|  | L120 | 32.72 | -24.33 | Limpopo | 0.353 | 26.2.2012 | 9:45 | 20 | 0.10 | 0.324±0.081 | 0.035±0.010 | 11.4±7.0 | 1.280±0.054 |
|  | L121 | 32.97 | -24.36 | Limpopo | 0.343 | 25.2.2012 | 7:45 | 16 | 0.19 | 0.270±0.030 | 0.021±0.004 | 6.3±3.7 | 1.247±0.040 |
|  | L124 | 32.4 | -24.59 | Limpopo | 0.402 | 24.2.2012 | 13:15 | 20 | 0.40 | 0.540±0.069 | 0.058±0.017 | 12.8±15.1 | 1.247±0.038 |
|  | L401 | 32.42 | -24.23 | Limpopo | 0.346 | 26.2.2012 | 9:00 | 20 | 0.05 | 0.420±0.056 | 0.042±0.008 | 14.9±6.3 | 1.211±0.054 |
|  |  |  |  |  |  |  |  |  |  |  |  |  |  |
| *N. orthonotus* | L034 | 32.82 | -22.15 | Limpopo-Chefu | 0.283 | 28.2.2012 | 10:45 | 14 | 0.87 | 0.416±0.170 | 0.007±0.006 | 0.1±0.3 | 1.419±NA |
|  | L124 | 32.4 | -24.59 | Limpopo-Chefu | 0.401 | 24.2.2012 | 14:00 | 22 | 0.81 | 0.762±0.207 | 0.062±0.018 | 1.3±4.1 | 1.181±0.128 |
|  | L249 | 36.92 | -17.82 | North Coast | 0.791 | 8.3.2012 | 8:30 | 21 | 0.19 | 0.194±0.055 | 0.016±0.010 | 4.9±4.3 | 1.175±0.041 |
|  | L304 | 33 | -24.35 | Limpopo-Chefu | 0.343 | 26.2.2012 | 15:00 | 19 | 0.89 | 1.209±0.264 | 0.076±0.019 | 0.9±3.9 | 1.162±0.046 |
|  | L358 | 34.87 | -21.03 | Central | 0.549 | 3.3.2012 | 13:30 | 16 | 0.56 | 0.512±0.104 | 0.048±0.017 | 7.1±9.7 | 1.175±0.031 |
|  | L401 | 32.42 | -24.23 | Limpopo-Chefu | 0.345 | 26.2.2012 | 8:45 | 9 | 0.22 | 0.721±0.161 | 0.041±0.018 | 9.3±6.9 | 1.165±0.111 |
|  | L414 | 32.73 | -22.55 | Limpopo-Chefu | 0.254 | 29.2.2012 | 9:30 | 10 | 0.5 | 1.223±0.787 | 0.038±0.033 | 2.9±4.4 | 1.278±0.059 |
|  | L423 | 33.92 | -22.08 | Limpopo-Chefu | 0.340 | 13.3.2012 | 11:00 | 20 | 0.5 | 1.693±0.545 | 0.04±0.0150 | 0.8±0.9 | 1.365±0.142 |
|  | L424 | 34.66 | -20.91 | Central | 0.547 | 12.3.2012 | 7:45 | 20 | 0.15 | 0.814±0.137 | 0.082±0.037 | 18.1±13.6 | 1.265±0.058 |
|  | L511 | 34.1 | -20.68 | Central | 0.511 | 4.3.2012 | 8:30 | 13 | 0 | 1.997±0.864 | 0.127±0.056 | 29.5±15.2 | 1.259±0.036 |
|  | L514 | 34.78 | -19.7 | North Coast | 0.819 | 5.3.2012 | 10:00 | 19 | 0.53 | 1.158±0.159 | 0.068±0.018 | 5.3±9.3 | 1.127±0.090 |
|  | L517 | 34.98 | -19.73 | North Coast | 0.903 | 5.3.2012 | 15:00 | 22 | 0.91 | 0.373±0.073 | 0.015±0.007 | 0.1±0.3 | 1.215±0.090 |
|  | L520 | 35.83 | -18.23 | North Coast | 0.547 | 7.3.2012 | 9:45 | 19 | 0.53 | 0.259±0.100 | 0.014±0.018 | 3.9±6.5 | 1.175±0.051 |
|  | L533 | 32.92 | -24.72 | Limpopo-Chefu | 0.439 | 1.3.2012 | 10:30 | 19 | 0.21 | 1.135±0.237 | 0.123±0.041 | 32.4±18.8 | 1.210±0.037 |

Table S2. Model-averaged coefficient estimates for predictors of Ovary Mass in *N. furzeri* (A) and *N. orthonotus* (B). Each estimate is based only on models containing the focal term, which prevents its shrinkage to zero (conditional model averaging) (Burnham & Anderson 2002). Relative Variable Importance is the sum of weights of those models containing the focal term. In *N. furzeri* (A), negative averaged coefficient estimate of the Sampling Time×Body Mass interaction indicates that larger females have lower relative Ovary Mass later during the day.

(A)

| Term | Estimate | SE | Relative Variable Importance |
| --- | --- | --- | --- |
| Intercept | 5.010 | 0.694 |  |
| **Body Mass** | **1.678** | **0.443** | **1.00** |
| Aridity Index | -2.023 | 2.391 | 0.61 |
| **Sampling Time** | **-0.000579** | **0.000333** | **0.98** |
| **Sampling Time**×**Body Mass** | **-0.000627** | **0.000232** | **0.86** |
| LIMPOPO | 0.108 | 0.239 | 0.35 |
| Aridity Index×Body Mass | -2.119 | 1.837 | 0.22 |
| LIMPOPO×Body Mass | 0.0166 | 0.208 | 0.09 |

(B)

| Term | Estimate | SE | Relative Variable Importance |
| --- | --- | --- | --- |
| Intercept | 3.364 | 0.600 |  |
| **Body Mass** | **1.341** | **0.307** | **1.00** |
| Aridity Index | 0.975 | 0.827 | 0.34 |
| CENTRAL | 0.195 | 0.497 | 0.36 |
| LIMPOPO-CHEFU | -0.438 | 0.416 | - |
| Sampling Time | 0.00013 | 0.000724 | 0.48 |
| CENTRAL×Body Mass | -0.572 | 0.320 | 0.17 |
| LIMPOPO-CHEFU×Body Mass | -0.611 | 0.253 | - |
| Aridity Index×Body Mass | 0.720 | 0.540 | 0.08 |
| Sampling Time×Body Mass | -0.000239 | 0.000507 | 0.11 |

Table S3. Coefficient estimates for predictors of Egg Size. Estimates of fixed effects coefficients from the best model (m5b, Akaike weight = 0.99) for *N. furzeri* populations sampled in 2011 (A) and calculated for the same model when 2012 data were applied (B). Model-averaged coefficient estimates of fixed effects for *N. orthonotus* (C). The estimate average is based only on models containing the focal term, which prevents its shrinkage to zero (conditional model averaging) (Burnham & Anderson 2002). Relative Variable Importance is the sum of weights of those models containing the focal term. The estimates for the coefficient of the interaction term Egg Size/fecundity trade-off are indicated in bold typeset. Apparently, in both years, the trade-off coefficient of Limpopo females deviates from zero, while is minimal in Chefu females making the trade-off with fecundity for Egg Size unimportant.

(A)

| Term | Estimate | SE |
| --- | --- | --- |
| CHEFU | 1.247 | 0.016 |
| LIMPOPO | 1.375 | 0.033 |
| Ovary Mass | 0.0114 | 0.00115 |
| Number of Eggs | -0.00131 | 0.000347 |
| Ovary Mass×Number of Eggs | 0.000635 | 0.0002.76 |
| Ovary Mass×LIMPOPO | 0.0309 | 0.0285 |
| Number of Eggs×LIMPOPO | -0.00325 | 0.000849 |
| Ovary Mass×Number of Eggs×LIMPOPO | 0.00317 | 0.00108 |

(B)

| Year | Mitochondrial lineage | Term | Estimate | SE |
| --- | --- | --- | --- | --- |
| 2011 |  | Random effects: |  |  |
|  |  | Population SD | 0.027 |  |
|  |  | Population/Female ID SD | 0.034 |  |
|  |  | Residual SD | 0.053 |  |
|  |  | Fixed effects: |  |  |
|  | Limpopo | Intercept | 1.374 | 0.035 |
|  |  | Ovary Mass | 0.0410 | 0.0267 |
|  |  | Number of Eggs | -0.00458 | 0.000793 |
|  |  | Ovary Mass×Number of Eggs | **0.00381** | **0.00107** |
|  | Chefu | Intercept | 1.248 | 0.017 |
|  |  | Ovary Mass | 0.014 | 0.0123 |
|  |  | Number of Eggs | -0.00127 | 0.0123 |
|  |  | Ovary Mass×Number of Eggs | **0.000592** | **0.000284** |
|  |  |  |  |  |
| 2012 |  | Random effects: |  |  |
|  |  | Population SD | 0.021 |  |
|  |  | Population/Female ID SD | 0.030 |  |
|  |  | Residual SD | 0.055 |  |
|  |  | Fixed effects: |  |  |
|  | Limpopo | Intercept | 1.324 | 0.021 |
|  |  | Ovary Mass | 0.0297 | 0.0211 |
|  |  | Number of Eggs | -0.00430 | 0.000761 |
|  |  | Ovary Mass×Number of Eggs | **0.00301** | **0.000934** |

(C)

| Term | Estimate | SE | Relative Variable Importance |
| --- | --- | --- | --- |
| NORTH COAST | 1.188 | 0.044 | 0.38 |
| CENTRAL | 1.286 | 0.040 | - |
| LIMPOPO-CHEFU | 1.275 | 0.033 | - |
| **Ovary Mass** | **-0.0141** | **0.0446** | **0.92** |
| **Number of Eggs** | **-0.00274** | **0.00161** | **0.92** |
| **Number of Eggs×Ovary Mass** | **0.00235** | **0.001.61** | **0.92** |
| Ardity Index | -0.186 | 0.129 | 0.51 |
| Aridity Index×Ovary Mass | 0.184 | 0.108 | 0.1 |
| Aridity Index×Number of Eggs | -0.00147 | 0.00619 | 0.1 |
| Aridity Index×Number of Eggs:Ovary Mass | -0.00403 | 0.00626 | 0.1 |
| CENTRAL×Ovary Mass | -0.0478 | 0.0539 | 0.01 |
| LIMPOPO-CHEFU×Ovary Mass | -0.0685 | 0.0504 | - |
| CENTRAL×Number of Eggs | 0.000383 | 0.00271 | 0.01 |
| LIMPOPO-CHEFU×Number of Eggs | 0.00111 | 0.00260 | - |
| CENTRAL×Number of Eggs×Ovary Mass | -0.0000691 | 0.00220 | 0.01 |
| LIMPOPO-CHEFU×Number of Eggs×Ovary Mass | 0.000547 | 0.00224 | - |
